# Rethinking the 'global' in global health: a dialectic approach

**DOI:** 10.1186/1744-8603-6-19

**Published:** 2010-10-28

**Authors:** Kayvan Bozorgmehr

**Affiliations:** 1 Department for International Health Sciences; Institute for Social Medicine, Epidemiology and Health Economics; Charité - University Medical Center, Berlin, Germany

## Abstract

**Background:**

Current definitions of 'global health' lack specificity about the term 'global'. This debate presents and discusses existing definitions of 'global health' and a common problem inherent therein. It aims to provide a way forward towards an understanding of 'global health' while avoiding redundancy. The attention is concentrated on the dialectics of different concepts of 'global' in their application to malnutrition; HIV, tuberculosis & malaria; and maternal mortality. Further attention is payed to normative objectives attached to 'global health' definitions and to paradoxes involved in attempts to define the field.

**Discussion:**

The manuscript identifies denotations of 'global' as 'worldwide', as 'transcending national boundaries' and as 'holistic'. A fourth concept of 'global' as 'supraterritorial' is presented and defined as 'links between the social determinants of health anywhere in the world'. The rhetorical power of the denotations impacts considerably on the object of 'global health', exemplified in the context of malnutrition; HIV, tuberculosis & malaria; and maternal mortality. The 'global' as 'worldwide', as 'transcending national boundaries' and as 'holistic' house contradictions which can be overcome by the fourth concept of 'global' as 'supraterritorial'. The 'global-local-relationship' inherent in the proposed concept coheres with influential anthropological and sociological views despite the use of different terminology. At the same time, it may be assembled with other views on 'global' or amend apparently conflicting ones. The author argues for detaching normative objectives from 'global health' definitions to avoid so called 'entanglement-problems'. Instead, it is argued that the proposed concept constitutes an un-euphemistical approach to describe the inherently politicised field of 'global health'.

**Summary:**

While global-as-worldwide and global-as-transcending-national-boundaries are misleading and produce redundancy with public and international health, global-as-supraterritorial provides 'new' objects for research, education and practice while avoiding redundancy. Linked with 'health' as a human right, this concept preserves the rhetorical power of the term 'global health' for more innovative forms of study, research and practice. The dialectic approach reveals that the contradictions involved in the different notions of the term 'global' are only of apparent nature and not exclusive, but have to be seen as complementary to each other if expected to be useful in the final step.

## Background

Last year in *The Lancet*, Koplan and his colleagues called for a common definition of 'global health' as being 'an area for study, research, and practice that places a priority on improving health and achieving equity in health for all people worldwide' [1]. In their article, they proposed several distinctions between public, international and global health and derived the above-mentioned definition from the geographical reach, level of cooperation, object and orientation of the different fields. Their manuscript posed some important questions, which are key to an understanding and conceptualisation of 'global health'.

However, the author of this manuscript argues that Koplan and his colleagues did not provide an adequate answer to one of the most crucial questions in attempts to conceptualise 'global health', which is: What is the 'global' in 'global health'?

The answer they provided to this question is that 'global' refers to any health issue 'that concerns many countries' or 'is affected by transnational determinants [..] or solutions'. They further state that the 'global' in 'global health' '[..] refers to the scope of the problems, not their location'. [1]

This definition of 'global', however, is imprecise, since it is unclear where the benchmark is in quantitative terms for the descriptor 'many countries'. Secondly, linking the term 'global' with the attribute 'transnational determinants [..] or solutions' does not present enough clarity about the difference to the object of the discipline 'international health' and is thus redundant. As such, efforts of the European Commission to harmonize health policies in the European Union would *per definition *become a 'global' health issue due to the 'transnational' character of any policies formulated by this institution; a certainly questionable consequence of this definition. Finally, the term 'scope of the problem' is highly inappropriate to define the 'global', since this attribute depends on subjective criteria rather than objective ones.

In the call for a common definition [1], not only is the term 'global' not very helpful to determine the object of the field 'global health', but it also does not legitimate the newness of a field complementary to 'international health' or 'public health'.

On the contrary: if 'global' is not accurately defined, the difference between 'global health' as a 'new' phenomenon and traditionally well-known influences on health remain sloppy. Furthermore, 'global health' as 'an area for study, research, and practice' is easily blurred with 'study, research and practice' in the fields of international or public health.

This conflict is reflected by the recent reaction of representatives of the 'public health' community, who promptly proclaimed in *The Lancet *that 'global health is public health', disagreeing with the attempt to distinguish between the fields. In their response [2] to the call for a common definition, Fried and her colleagues illustrate that 'global health and public health are indistinguishable' [2] based on the criteria they present [1]. They further stress - perhaps correctly - that the attempt to *distinguish differences *between 'global health' and 'public health' conflicts with the key tenets of a 'global public health' strategy [2].

Similar reactions might occur from representatives of the 'international health' community, contending that most of what is labelled as 'global health' today is an original domain of their field. This is only a matter of time given the fact that many of the 'global health programmes' that are mushrooming, for instance, in the United States in the field of education, are merely re-labeled uni- or bi-directional exchange programmes between two countries [3], which were previously called 'international health programmes'.

Important to note is that the discussion about the descriptor 'global' in 'global health' is not an academic one, leading into the ivory tower. It is a crucial point for identifying and setting priorities for educators, researchers and practitioners in the field of 'global health'. An accurate understanding of the 'global' in 'global health' is the prerequisite to answer the key questions posed by Koplan and colleagues *without *raising conflicts with other fields or producing redundancy. In particular, being clear about this term is necessary to determine what exactly makes a health problem, determinant or solution (or a component of it) 'global'. Finally, it avoids that impreciseness and confusion discredits the importance of 'global health' as an analytical or practical category.

But, what exactly is 'global' about 'global health'?

The following paragraphs define the term 'health' in 'global health', present existing definitions of 'global health' and a common problem inherent therein. In a next step, the author presents different denotations of the term 'global' in 'global health' and applies these to the areas of malnutrition; HIV, tuberculosis & malaria; and maternal mortality. This procedure depicts the dialectics involved in the term and illustrates how these impact on the object of 'global health' as an area of study, research and practice. The debate continues by putting the proposed concept of 'global' in context with other views on 'global' and 'local'. It then closes with reflections on (i) normative objectives attached to 'global health' definitions and (ii) paradoxes involved in attempts to define 'global health'. The author thereby hopes to provoke further debate and intellectual energy spent on this topic.

The dialectic approach (to re-thinking the 'global' in 'global health') hereby refers to a mechanism of rational validation [4], i.e. to a process in which contradictions in given concepts or hypotheses are revealed. Bringing to light the contradictions thus leads to their withdrawal (i.e. of the concepts/hypotheses) as (sole) candidates for knowledge generation and (ideally) to the acceptance of other concepts or hypotheses. The latter ones (ideally) overcome the apparent contradiction at one level by integrating a synthesis of the opposing poles at a higher level of conceptual analysis.

## Discussion

### The 'health' in global health

Since health is understood as physical, mental and social wellbeing and not merely as the absence of disease [5], it is clear that 'global health' does not mean 'the absence of disease worldwide'. Therefore, whatever 'global' health is, it is more than an engagement with diseases on a worldwide scale; and thus more than the aggregation of data, indicators, mortality or morbidity on a global (*read: worldwide*) scale.

While information gathered globally (*read: worldwide*) can help to open insights into the worldwide distribution and burden of diseases, the object of the field 'global health' has to go beyond that.

Accepting, in addition to the above, that health is a social, economic and political issue as well as a fundamental human right [6], helps to pave the way to an object of the field beyond diseases and 'disease burden'. This understanding of 'health' in 'global health' does not only do justice to the upscale and importance, which the social determinants of health have recently received on the health agenda globally (*read: worldwide*) [7], but also provides a useful approach to conceptualise the field of 'global health' in research, education and practice beyond bio-medical approaches.

But: what is the difference between other health-related fields, such as 'public health' or 'international health', which are concerned with these influences on health?

### Global health - the definition problem

The newly coined term 'global health' reflects the attempt to differentiate the concerns of 'global health' from the traditional focus of interest associated with the term 'international health' [8]. This discipline which roots back to the era of colonisation of the 'new worlds' concentrates predominantly on infectious diseases and related tropical medicine in developing countries [9,10].

Although several definitions of 'global health' are currently under discussion, this field is generally employed under a more embracing concept, i.e. 'health problems, issues and concerns that transcend national boundaries [..] and are best addressed by cooperative actions and solutions' [11]. Other definitions rather focus on concerns and determinants of health that are beyond the control of national states and their institutions [12] or are affected by globalization and therefore subject to institutions of 'global health governance' [13].

Stuckler and McKee, in contrast, use different metaphors to describe 'global health' in the field of policies. These range from 'global health' as foreign policy, as security, as investment or as charity to 'global health' as 'public health' issue [14]. Although this approach depicts important perceptions of the term among different actors in 'global health', it is important to note that metaphors are not definitions. As such, the metaphor 'global health-as-XYZ' does not describe anything which is not expressible through pre-existing vocabulary. Rather, it raises the question of why we need a term called 'global health', which implies and subsumes all these different meanings and literally becomes a 'one-term-fits-all'?

Dodgson and his colleagues on the other hand define a 'global health issue' very broadly as 'one where the actions of a party in one part of the world can have widespread consequences in other parts of the world' [15].

Rowson and his collaborators formulate an encompassing and yet unpublished definition of global health in the year 2007. As is pointed out in the following paragraph, their definition brings key aspects of the above mentioned definitions of 'global health' and 'health' together:

"Global health is a field of **practice, research and education **focussed on health and the **social, economic, political and cultural forces **that shape it across the world. The discipline has an historical association with the **distinct needs of developing countries **but it is also concerned with health-related issues that **transcend national boundaries **and the differential **impacts of globalisation**. It is a **cross-disciplinary field**, blending perspectives from the natural and social sciences to understand the social relationships, biological processes and technologies that contribute to the improvement of health worldwide." [8]

This definition includes the developing country heritage of the term 'international health' as well as the new emphasis on the impacts of globalization, including on industrialized countries. At the same time, Rowson and colleagues offer some clarity about the object of 'global health' and the types of knowledge required to practice this field. Similar to the definition published in *The Lancet *[1], they widen the horizon of 'global health' from practice into the areas of research and education as a cross-disciplinary field, which builds upon methods from public and international health sciences. The outcome of an engagement in the field of 'global health', according to the above, is the understanding of various social, biological and technological relationships that contribute to health improvements worldwide. Koplan and colleagues, on the other hand, placed an additional 'priority on improving health and achieving equity in health for all people worldwide' as an objective of engaging in 'global health'.

Notably, the commonality in all of the above definitions, including the metaphors, is that the term 'global' is not straightforwardly defined. Rather, it seems that 'global' in 'global health' is apparently regarded either in terms of 'worldwide' or 'issues that transcend national boundaries'.

A view at the recent scholarship on the interface between anthropology and 'global health' reveals further notions of 'global'. In a stimulating article, Janes and Corbett draw upon different understandings of 'local' and 'global' and propose the following definition of 'global health' as it pertains to anthropology: 'Global health is an area of research and practice that endeavours to link health [..] to assemblages of global processes [..]' [16]. The 'global' used here does not only mean 'worldwide' in a spatial dimension, but also refers to 'phenomena as having a "global" quality' [17] (*p.10*). That is, to 'phenomena whose significance and validity are not dependent on the 'props' of a 'culture' or a 'society'' (*ibid., p.10*) and thus can, for example, include biological life on the planet itself. The term 'assemblage' in the definition refers to unstable, forming or shifting products of 'multiple determinations that are not reducible to a single logic' or to a 'locality' (*ibid., p.12*) (For other usage of the term assemblage see [18,19]). As for health, Janes and Corbett note that both 'theoretically and methodologically the task is to understand how various assemblages of global, national, and subnational factors converge on a health issue, problem, or outcome in a particular local context' [16].

This definition builds upon a denotation of 'global' referred to, as we proceed, as the 'holistic' approach.

The next paragraphs, however, will show that the above denotations alone are of limited use. Arguments and analysis that build on these conceptions alone either fail to open insights that are not available through pre-existent vocabulary or entail analytical problems and overlaps. As such, the problem to distinguish the object of the field 'global health' from those of international and public health sciences is not resolved.

### Denotations of the term 'global'

As presented above, the 'global' in 'global health' can be understood in different ways. Firstly, 'global' can mean 'worldwide', 'everywhere' and stand for a universally prevalent agent. Secondly, the 'global' can refer to 'issues that transcend national boundaries'. Thirdly, it can imply a 'holistic' denotation, referring to *all *and *everything *which impacts on health, ranging from biological, molecular levels to 'higher' (or other) levels by building complex 'assemblages' (*'higher' is hyphenated since the author does not attempt to attribute scale to 'levels' in terms of micro-macro binaries*).

However, there is a fourth way to conceptualise the 'global' that considerably differs from the above-mentioned concepts. Acknowledging that globalization is the motor of the evolution of the term 'global health' (as pointed out by both the definitions of Kickbush [13] and Rowson and colleagues [8]), the author suggests that a stronger engagement with the same paves the way to a more innovative understanding of 'global' in 'global health'.

#### Global as supraterritorial

The globalization process in contemporary history involves the spread of 'reductions in barriers to transworld contacts' and has thus enabled people to become physically, legally, culturally, and psychologically engaged with each other in 'one world'. Through these reductions, the global sphere has become a social space in its own right and is not any more simply a collection of smaller geographical units like nations, countries and regions, but rather a spatial unit itself. [20] New in contemporary history in this context is the rise of 'globality', which entails the large scale spread of 'supraterritorial' processes and connections, whose impacts nevertheless always 'touch down' in territorial localities.

According to Scholte, 'supraterritorial' relations are *social connections *that transcend territorial geography, understood as domains mapped on the land surface of the earth, plotted on the three axes of longitude, latitude and altitude.

For example, 'developments such as climate change, stratospheric ozone depletion, pandemics, and losses of biological diversity unfold simultaneously on a world scale. They envelop the planet at one place at one time; their causes and consequences cannot be divided and distributed between territorial units'.

Thus, **globality refers to 'social links between people located at points *anywhere *on earth**, within a whole-world context'. [20] While globalization becomes a reconfiguration of social space, the term 'supraterritoriality' describes this evolving shift.

Before applying this concept of the 'global' on health, it is crucial to note the following five aspects emphasised by Scholte regarding the 'global-local-relationship' inherent in global-as-supraterritorial:

1. Today's world is both territorial *and *supraterritorial, i.e. the addition of supraterritiorial qualities of geography has not eliminated the territorial aspects: territorial relations are no longer purely territorial, and supraterritorial relations are not wholly un-territorial. Contemporary society knows no pure globality that exists *independently *of territorial spaces, which means that the 'present world is globalizing, not totally globalized'.

2. While it is helpful to distinguish different spheres of social space, the global (*read: supraterritorial*) is not a domain unto itself, separate from the regional, the national, the local, the community or the household. For example, a government may be sited at a national (*read: territorial*) 'level', but it is a place where both supraterritorial and inter- or trans-territorial spaces converge.

3. A social condition is not positive or negative according to whether it is local (*read: territorial*) or global (*read: supraterritorial*) and local/global polarizations which depict the local as *immediate *and intimate and the global as *distant *and isolating are neither useful nor hold up to closer scrutiny.

4. Globality links people *anywhere *in the world, but it does not follow that it connects people *everywhere*, or to the same degree. That means there are variations in the extent of supraterritoriality and transworld connectivity along territorial positions (e.g. in North America, Western Europe and East Asia more than in other world regions; across urban lines more than across rural) or along social positions (the wealthier accessing more transworld contacts than the poor).

5. Finally, social space *always *involves politics: processes of acquiring, distributing and exercising social power. Transworld and supraterritorial connections invariably house power relations and associated power struggles, whether latent or overt. Global (*read: supraterritorial*) links are venues of conflict and cooperation, hierarchy and equality, opportunity and its denial. [20]

### The dialectics of the term 'global'

#### Applying the 'global' to health

In Additional File 1, the different concepts of 'global' presented above have been applied to the areas of i) malnutrition, including over- and undernutrition, ii) HIV, tuberculosis & malaria and iii) maternal mortality in order to exemplify how the different concepts impact on the object of the field. Thereby, it is possible to reflect on the applicability and adequacy of the different concepts.

This procedure (see Additional file 1) reveals the dialectics involved in the different concepts. It illustrates that denotations of 'global' as 'worldwide', 'everywhere', 'universal' or as 'transcending national borders' (alone) are of limited use for attempts to produce new knowledge or to present new objects for research, education or practice. How come?

#### Applying 'global-as-worldwide' to health

The 'global-as-worldwide' is misleading and, where applicable (i.e. where health problems show a really 'universal' prevalence), highly redundant to 'public health'. This is shown in the example of overnutrition. With 'global-as-worldwide', overnutrition or obesity becomes a 'global' health issue, since it is a worldwide (public) health problem. The problem can be found globally (*read: worldwide*) to different extents [21], either among better-off or among socio-economically disadvantaged classes. Thus it can be considered as a global (*read: worldwide or universal*) health risk [22]. In this context, however, representatives of the public health community can correctly argue, that issues of food, nutrition, eating habits and physical activity are traditional fields of their work in research, education and practice.

On the other hand, the concept is misleading, because the rhetoric of *worldwide *does not legitimate calling health challenges that are confined on particular regions or continents (*read: territorial units*) to be called 'global health problems'. This is the case for undernutrition, malaria or maternal mortality (see Additional file 1), since, for example, 95.0% of maternal deaths worldwide are concentrated in sub-Saharan Africa and Asia [23].

It is also misleading in the sense that, if following the logic of 'global-as-worldwide' - while being consciously polemic - ambitioned dermatologists could soon proclaim tinea pedis as the next global (*read: worldwide*) health problem.

#### Applying 'global-as-transcending-national-boundaries' to health

With 'global-as-transcending-national-boundaries', neither overnutrition nor undernutrition nor any other non-communicable diseases are *directly *'global' health issues. Rather, the carriers and determinants that transport risk factors and lifestyles across *more than one *country and lead to malnutrition, for example international trade, become the object of 'global health'.

Well known, however, is the fact that intensified trade gave rise to the International Sanitary Conferences in 1851 and thus to the birth of the international (public) health era [24]. This era brought about a great quantity and diversity of international legal regimes on global (*read: universal- and/or transcending-national-boundaries*) health risks [24]. Therefore it is questionable whether it is legitimate to declare international trade an object of 'global health', only because today trade is intensified globally (*read: worldwide*).

Furthermore, with 'global-as-transcending-national-boundaries' all communicable diseases *per se *and all determinants affecting *more than one country *(i.e. transcend at least *one *national border) become the object of 'global health'. Without any benchmarks about how many borders an issue needs to transcend to become 'global', this concept causes high redundancy with the object of 'international health'. In this context, it is not worth mentioning that such benchmarks would be more than inappropriate.

#### Applying 'global-as-holistic' to health

Similarly, a 'holistic' understanding of the 'global' in 'global health', which includes *all *influences on health on molecular, individual, regional, national, international and global (*read: worldwide or transcending national boundaries*) levels (see Additional file 1) is an analytical dead-end. An approach to deal with *all *influences on health on *all *levels is deeply unsatisfactory for serious social analysis and the policy decisions, descriptions, explanations, evaluations, prescriptions and actions that result from it. No doubt, the term 'global assemblage' [16,17] is a useful metaphor to illustrate the complexity of today's world and its health determinants. But, using this 'holistic' concept as the level of analysis means that every determinant in question (be it a particular policy, a crisis, etc.) literally 'falls' into and becomes part of a 'sea of forces' produced by other health determinants. The health outcome, viz. the influence or impact on health, is thus a *function of the vector *produced by *all *forces. Any particular analysis thus entails the question of how wide to span the 'vector space'. One could think of distinguishing 'positive' and 'negative' constellations of 'assemblages'. 'Positive' constellations would be those that change the direction of the vector-bundle towards 'good health' and 'negative' ones would have the opposite 'effect' at the *ultimate level *of the individual/household/population. The important *entry points *and *pathways *of (as well as interactions *between*) the single 'positive' and 'negative' vectors *before *'reaching' the ultimate level, however, remain (from the author's point of view) a 'black box'. The problem of 'organizing the evidence into a coherent story' by building the evidence up 'link by link' [25] is not solved if the 'global' itself represents the 'whole picture'.

#### Applying 'global-as-supraterritorial' to health

On the other hand, the concept of 'global-as-supraterritorial' adds 'new' objects to existing health related disciplines. With this concept, diseases and illnesses remain what they have been before, that is either medical, public or international health problems; or all of them. The disease specific aspects, however, become symptoms of underlying structural determinants *AND *their supraterritorial links. The object of 'global health', with global-as-supraterritorial, is the analysis of the 'new' social space created by globalization. **Globality, in the context of health, then refers to supraterritorial links between the social determinants of health located at points *anywhere *on earth**. As such, representatives of the medical, the public health, or the international health community can engage in 'global health' education, research or practice without producing redundancy. Building on the generic expertise of their field, representatives of those communities - or the health workforce in general - can broaden their focus towards 'global health'. They can impart and gain knowledge, produce new insights, or develop solutions related to global (*read: supraterritorial*) links between the social determinants of health, which are in themselves global (*read: universal*) determinants.

**Figure 1:**
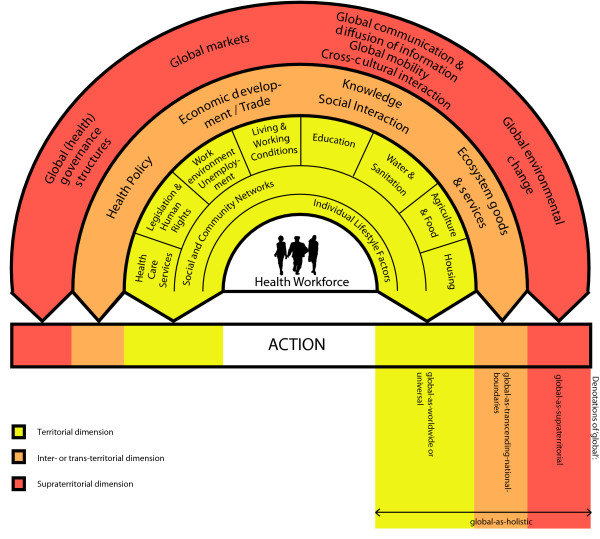
**Concept of global health**. Territorial dimension: includes for example determinants on territorial units such as community upto state or national units; Inter- or trans-territorial dimension: includes for example determinants which link and/or transcend territorial units, e.g. national borders; Supraterritorial dimension: includes social, political, economic and cultural links between determinants of health anywhere in the world regardless of territory in terms of geography.

The interaction of the health workforce with the deduced object of the field is illustrated in a concept of 'global health' in Figure 1, which was originally produced as a framework to assess 'global health' in the field of education in Germany [26]. This concept is adapted from and builds upon the 'social determinants of health model' of Dahlgren and Whitehead [27] and a model of 'globalisation and health' of Huynen and colleagues [28]. These models schematically separate determinants of health in layers, beginning with individual and 'proximal' determinants of health and reaching more 'distant' layers. It is crucial to note, however, that with the above definition of 'global-as-supraterritorial', the 'distant' layers are not 'distant'. Instead, 'global' (*read: supraterritorial*) layers link the determinants of health horizontally *anywhere *in the world and impact on them through complex pathways, while being influenced by the same or other determinants in a mutual relationship.

The following underpins the applicability of the concept of 'global-as-supraterritorial' to health, particularly related to the aspects emphasised by Scholte (see above notes 1- 4):

In the context of HIV, malaria and tuberculosis, access to essential medicines is a global (*read: universal*) determinant of health and a major public or international health concern. With 'global health' focusing on the supraterritorial links between this determinant anywhere in the world, the object becomes inevitably linked with international agreements and trade regimes, such as the Trade-Related Aspects of Intellectual Property Rights (TRIPS). This agreement, formulated by the World Trade Organization (WTO) as an international (*read: interterritorial*) organization and signed by national (*read: territorial*) governments, has a global (*read: supraterritorial*) character, since it links the determinant 'access to medicines' *anywhere *in the world (i.e. in the 153 countries which have signed up to the WTO), but not *everywhere *in the world (for example not yet in least developed countries).

In the context of maternal mortality (MM), while global-as-worldwide was not capable of creating 'new' objects for research, education or practice, the concept of global-as-supraterritorial creates interesting and powerful ones (see Additional file 1) for analysis, teaching or action for the 'global health community'. Some examples from the literature are: the role of global (*read: supraterritorial) *institutions in impeding [29] or catalysing efforts to control MM; the impacts of the global (*read: worldwide and supraterritorial*) food and economic crises on the determinants of MM, such as nutrition, diet and food availability [30]; the role of territorial policies with supraterritorial impact on shortages of health professionals [31,32] and thus on quality of care; or legal frameworks and human rights connections of the determinants of MM [33].

**Figure 2:**
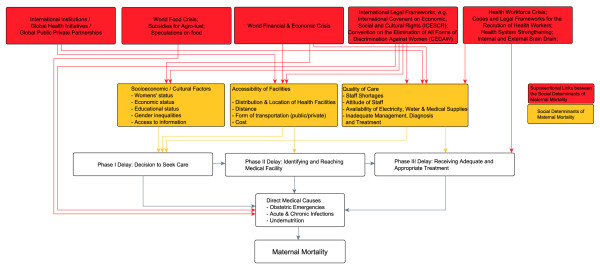
**Supraterritorial links between the Social Determinants of Maternal Mortality**.

The interplay of selected supraterritorial links between the social determinants of MM is illustrated in simplified form in Figure 2. While the major direct causes of MM in developing countries, such as haemorrhage and hypertensive disorders [34], are preventable by timely direct medical treatment, the causes known to influence the delay in seeking, reaching and receiving care [35] are also objects of supraterritorial influences, which can be seen as the *causes of the causes *of delay (Figure 2). With global-as-supraterritorial, the 'global-health-part' of MM are the *social links between the underlying structural determinants of maternal health anywhere in the world*. As such, the magnitude of MM rates becomes a symptom of these direct and indirect influences on maternal health and a *starting point *to learn about, research on or act upon these influences (Figure 2).

This concept adds 'new', namely non-redundant, objects to conventional approaches that analyse maternal mortality via 'global health' concepts with global-as-worldwide or -as-transcending-national-boundaries. It produces 'clearer', namely more distinct, objects compared to concepts building on global-as-holistic (see Additional file 1).

#### Applying 'global-as-supraterritorial' to health in other contexts

Of course, the 'holistic' approach (Figure 1) allows for (consciously or unconsciously) 'see-sawing' between *all *concepts. This switch of concepts can be observed, for example, when Janes and Corbett explicate key-arenas of research and practice at the interface of 'global health' and anthropology [16]. While following their line of arguments one realizes that they switch between global-as-worldwide, global-as-transcending-national-borders, and (what has been described here as) global-as-supraterritorial - whether they are always aware of this fact or not. In light of their definition of 'global health' (see above or [16]), switching between different concepts is completely legitimate and highly inclusive. At the same time, however, the flexibility constitutes the Achilles' tendon of their definition. This soft spot offers a contact point for the same strong critique invoked by Fried and her colleagues, arguing that original fields of 'public health' are repackaged into 'global health' [2]. As an example: the described conflict *could *erupt when Janes and Corbett (2009) argue that anthropologists' contribution in the field of 'global health' would be to explicate or ground 'health inequities in reference to upstream constellations of international political economy, regional history, and development ideology' [16]*(p.170)*. Beyond doubt, all contributions cited by them in this particular context have their merit and importance in, what they call, 'exposing processes by which people are constrained or victimized or resisting external forces in the context of local social worlds' *(ibid)*. Nevertheless, the engagement with these unspecified upstream constellations could also pertain to a critical 'public health' discipline, conceptualised as an equity focussed, investigative and confronting discipline, aimed at improving the lives of the vulnerable by identifying, mitigating or opposing structural violence on 'local social worlds'.

On the contrary, a 'global health' approach that *consciously *and *explicitly *applies the concept of global-as-supraterritorial would focus on exposing *the links *between processes by which people *anywhere *in the world 'are constrained or victimized or resisting external forces'. An important part of the force of this specification would be that the 'global-health-part' of explaining health inequities [16] would, firstly, not completely overlap with public health or other disciplines. Secondly, it would move the view of the 'global health community' *per definition *on to the burning (supraterritorial) issues, which Janes and Corbett indentify in their 'key-arenas' (such as ecosocial epidemiology, climate change, circulation of science and technology, pharmaceutical governance, patent protection or the power of consultancy agencies) [16].

Another exercise of re-thinking the 'global' demonstrates the applicability of the proposed concept. Apply the global-as-supraterritorial in context with the notion of 'inherently global health issues' (IHGIs), a term coined by Labonte and Spiegel [36]. Now ask yourself, both in light of all the above *and *the reasoning presented for IGHIs [36], *why *the issues presented there could be regarded as 'global' health issues.

The issues are indeed IGHIs (see also under "Global as supraterritorial"), but *not only *because of their inherent quality of being of 'universal' importance for people everywhere or *worldwide*. Also not because of their ability to 'transcend national borders' [36], which again entails the how-many-borders-question (leading to nowhere). More specifically, and less redundantly, it is because the IGHIs either constitute, house or draw our attention to *distinct links *between social determinants of people's health anywhere in the world.

In this context, it is worthwhile to have a look at Labonte and Torgerson's complex framework for health impacts of globalization (see Figure 2 in [25]), in which the IGHIs extend from household to global 'levels'. Their illustration of the framework indicates that they also attribute to the IGHIs the 'holistic' concept, including the (ambiguous) quality of local-global-simultaneity (on this quality see above notes 1-5 and the below '*Reflections on global-local- and global-global-relationships*'). But even with this reasoning we are again at the same point of discussion: the global-as-universal, -as-transcending-national-borders, or -as-holistic alone does not allay the critique invoked by a critical 'public health' (or 'international health') discipline claiming to be coequally concerned with IGHIs. Introducing the 'supraterritorial' in the analysis of pathways towards the territorial manifestations (e.g. towards 'water shortage'; 'war and conflict' [36]) can, however, legitimate the 'newness' of 'global health' as a field. It can unite different disciplines in analysing these links, namely the supraterritorial part of the IGHIs (for example: virtual water in 'water shortage'; the military-industrial-academic complex or arms trade in 'war and conflict').

Admittedly, the concept of global-as-supraterritorrial is very close to global-as-transcending-national-boundaries (see Additional file 1). In contrast to the latter concept, however, the 'supraterritorial' is more specific about the character of the process and does not cause redundance with inter- or trans-nationality by falling back into methodological territorialism. Methodological territorialism here means getting caught in the trap of thinking in pure geographic terms, e.g. in national units only [20]. By avoiding this, health policies in the European Union (see Introduction) remain transterritorial policies as long as they influence the determinants of health *in a specific transnational territory*; and do not become global (*read: supraterritorial*) ones per definition as long as the health policies do not link determinants *anywhere *in the world.

From all the above-mentioned definitions of 'global health', the character of global-as-supraterritorial is most closely aligned to the above definition of the agent described by Dodgson and colleagues, which makes an issue a 'global health issue' [15]. It is also close to Spiegel and Labonte's notion of 'globalization as determinant of health determinants' [37]. However, with globality as the supraterritorial link between the social determinants of health located at points *anywhere *on earth, this agent and the notion of 'globalization' receive more substance for researchers and educators in the field of 'global health'.

#### Global-as-supraterritorial in light of other views on 'global' and 'local'

This section aims to put the proposed concept of global-as-supraterritorial in context with selected influential works, dealing with the complexity and diversity of what is regarded to constitute 'global' and/or 'local' [17,38-45]. This undertaking opens far more chapters than can be addressed here in depth and as such does not claim to be exhaustive (for more comprehensive reviews see [40,45]).

The section is specifically concerned with the following two questions:

1. Does the global-local-relationship inherent in global-as-supraterritorial (see above notes 1 - 5) cohere or collide with other views on this relationship?

2. Does the 'global' in global-as supraterritorial cohere or conflict with other views of 'global'?

#### Reflections on global-local-relationships

##### 1.1 Cohering views: the global as produced in the local

Studies in the fields of anthropology [41] and sociology [42] have applied and provided useful concepts in this context. Building on attempts to 'ground globalization' along the three axes of 'global forces', 'global connections' and 'global imagination', Burawoy stresses that 'globalization is produced' in 'real organizations, institutions and communities' and is thus 'manufactured' [43]. He emphasises the *ambiguous *character of the 'global' by noting that '[w]hat we understand to be 'global' is itself constituted within the local; it emanates from very specific agencies [..] whose processes can be observed first-hand' [43]. According to Burawoy, the 'local' does not oppose the 'global'. Rather, globalization is produced through a chain of connections and disconnections, 'a local connected to other locales' [43]. Similar to Scholte, he thereby rejects global-local antinomies (see above notes 1 - 5). By stating that the connections all look 'different from different nodes in the chain' [43], he also emphasises another important issue, namely the position-dependence of observations and the importance of the perspective from which we look at or evaluate the 'global as produced in the local'. 'The same phenomenon can look like anti-politics from within the international agency, like political paralysis from within the state, like a social movement from the ground' [43]. The issue of position-dependence is central to the further debate on 'objectivity' in this manuscript and will be taken up again in reflections on normative objectives.

The above is also in line with Ginsburg and Rapp's understanding of 'the local' (also invoked by Janes and Corbett [16]). Their understanding of this term 'is not defined by geographical boundaries but is understood as any small-scale arena in which social meanings are informed and adjusted through negotiated, face-to-face interaction.' [41] (*p.8*) (for a critique of the 'face-to-face' definition of 'local' see [44]). By stating that 'transnational or global processes are those through which specific arenas of knowledge and power escape the communities of their creation to be embraced by or imposed on people beyond those communities' (*ibid., p.8-9*), they acknowledge that decisions, made in these 'local' arenas, may have 'drastically different' consequences in magnitude and/or spatial impact. This sense of 'local', although not defined by pure geography in form of national or subnational units, has undoubtedly a territorial quality. Decisions made locally can either have only local (*read: *territorial) or both local *and *global (*read: supraterritorial*) impacts. To apply the proposed terminology: decisions, made on Ginsburg and Rapp's 'small-scale arenas', on 'the local' [41] or on the 'territorial' [20] must not necessarily, but can influence people's social determinants of health *anywhere *in the world. In this case, the decisions themselves, the particular processes, institutions, agencies, legal frameworks and channels through which they are translated, realised, established or imposed constitute the *supraterritorial link *and thus the 'global' in 'global health'.

Framing these links as (random) 'assemblages' might produce somewhat misleading associations, since they are not *passively *assembled. These links and their operational channels and pathways are *actively *constructed, planned, governed and maintained. They are 'manufactured' [43] by social actors, formed and coined by their interests, motives and values. These links should be regarded as the 'global' in 'global health' and need the attention of researchers, educators and practitioners.

The *ambiguity *of the 'global' as being both territorial *and *supraterritorial clarifies how 'local' engagement in 'global' health can be possible.

##### 1.2 Colliding views: abstain from using global/local terminologies

Global-local antinomies and micro-macro binaries are also rejected by Latour [44], who - from the perspective of Actor-Network Theory (*see p.179*) - argues to 'localize the global' and 'redistribute the local' *(p.192-3)*. Thus, he draws our attention, firstly, at the 'connectors' that will '[..] only then, be allowed to freely circulate without ever stopping at a place called 'context' or 'interaction'' *(p.192-3) *and, secondly, at 'what is being transported: information, traces, goods, plans, formats, templates, linkages, and so on' *(p.204-5)*. Marcus, from an anthropological perspective, also places an emphasis on 'connections' when he argues that '[f]or ethnography, there is no global in the local-global contrast now so frequently evoked. The global is an emergent dimension of arguing about the connection among sites [..]' [38]. Latour's axiomatic argument that '[n]o place dominates enough to be global and no place is self-contained enough to be local' *(p.204) *is - in contrast to Burawoy's and Scholte's argumentation - invoked as a plea for abstaining 'from ever using the local/global [..] repertoire' *(p.206)*.

As such, his call to keep the social flat *(p.165-191) *inherently conflicts with Figure 1 and the term 'supraterritorial', because the term implies that something distant exists 'above/higher' given territories. This is especially the case if the above notes 1 - 5 are not *actively *kept in mind in this context. Recalling that the 'supraterritorial' is understood as 'social links between people anywhere in the world' [20], or (as proposed in the context of health) as links between social determinants of people's health anywhere in the world, might ease this (apparent?) conflict.

The following example illustrates this point. Although the above-described social sphere of global-as-supraterritorial seems to be quite 'distant' at the first glance for health professionals (Figure 1), this is not the case after closer scrutiny: there is an international (*read: interterritorial*) spread of local (*read: territorial*) efforts and initiatives to increase 'access to essential medicines' across Asia, Africa, Australia and Europe, as, for example, reflected by the many chapters of the Universities Allied for Essential Medicines [46]. Their actions can influence the 'supraterritorial' aspect of the determinant 'access to essential medicines' by framing 'knowledge' as a global (*read: universal*) public good. As such, local initiatives or their produced ideas [47,48] can shape or re-frame a global (*read: supraterritorial*) social space by influencing or adding to existing determinants and solutions. Supraterritorial associations of locally (*read: territorially*) working civil-society organisations can impact on determinants of health locally and at the same time influence determinants globally (*read: supraterritorially*), but not necessarily *worldwide or everywhere*.

Thus, in response to the first question addressed by this section: the 'global-local-relationship' inherent in the concept of global-as-supraterritorial [20] coheres with some anthropological and sociological views despite the use of different terminology [16,41-43]. But it (apparently?) conflicts with others [38,44] due to the same, if the emphasis on 'social links' is not actively kept in mind. Where coherence can be found [16,20,41-43], the authors argue - in Scholte's words - that 'local sites' can be territorial *and *supraterritorial at the same time (namely when they constitute or produce social links between people anywhere in the world).

#### Reflections on global-global-relationships

So what about the second question, which is concerned with other views on 'global'? The above section titled '*Applying the 'global' to health*' has already shown that (i) the global-as-supraterritorial collides with notions of global-as-worldwide and -as-transcending-national-boundaries, but (ii) can be seen as an element of global-as-holistic, or as 'assemblages' (see [16,17]).

##### 2.1 (Apparently) conflicting views: No de-territorialisation without re-territorialisation

Further notions describe the phenomenon of territorialisation, de-territorialisation and re-territorialisation elsewhere as 'transversal' movement (see [18] and [49] cited in [50] or in [45]). The term refers to a 'movement' that takes place between the intra- and interstate and extends into different political spaces. By invoking this term, Debrix uses an example of the international aid machinery to illustrate how organisations occupy and chart new 'territories', while escaping the spatial confines (e.g. the nation state) in which they were previously located. Territory seems here to be used both in terms of 'social space' *and *pure geography. Calling the Charter of Medecines Sans Frontières (MSF) an 'onto-territorial moment' that is creative of space, he follows the 'transversality' of MSF during their '[..] deterritorialisation (escaping the territory, the State apparatus) and reterritorialisation (marking new spaces and identities along new lines)' [50].

If we accept that every de-territorialisation calls for a re-territorialisation ([18] cited in [50]), one wonders what happens in between these two conditions? In this context, the descriptor 'supraterritorial' can be helpful, as the social space where 'transversal movements' take place after they de-territorialise (in terms of geography) and before they re-territorialise (in terms of geography and/or social space).

Due to the ambiguity and simultaneity of the global-local-relationship inherent in global-as-supraterritorial (see above notes 1 - 5), the supraterritorial space can take a bridging function between the relativist binaries, which are local-as-purely-territorial (or un-global) and global-as-purely-un-territorial (or un-local).

So Debrix is right when he concludes in his analysis of MSF's work that 'sociopolitical inclusions and exclusions, and the redistribution of power and knowledge [..], are inherent to spatial strategies even when they pretend to take on a *de-*territorial appearance' *(emphasis and hyphen added)*. But between the binaries of 'territorial' and '*de-*territorial' is what can be understood as the 'global', the 'supraterritorial', the social link between people anywhere in the world, which is not completely bound to territory *in terms of geography*, but is also not detached completely from the same, especially not *in terms of social space*. Debrix thus describes a social space, *supra*territorial, not *de*-territorial, created by MSF. This space constitutes a link between people's social determinants of health - namely access to health care - anywhere in the world.

##### 2.2 Complementary views: flows and '-scapes'

Invoking Appadurai's notion of '-scapes' (ethno-, media-, techno-, finance-, and ideoscapes) [51] at this place can even help to striate or specify particular social spaces in the yet 'empty' supraterritorial *land*scape and help to overcome this conflicting constellation (*supra*territorial vs. *land*scape). This notion has been used by Spiegel and colleagues to specify 'globalization' in its interaction with population health (see Figure 1 in [37]). Focusing on 'flows', the notion has been criticised for being theoretically too *detached *from political economy [45] and, in the author's view, seems detached from views of the 'global' as produced in the 'local' [40,43]. Bringing both notions (global-as-supraterritorial and xy-scapes) together, however, helps to attach the '-scapes' in a political social space (see above note 5).

In the case of Debrix's example of the international aid machinery, the 'transversal movements' take place by creating 'victimhood' [50], by using (or becoming part of) mediascapes, by feeding the ideoscapes with ideas of humanitarianism, by exercising power in identifying who is the victim and who not and thereby linking the determinant 'access to health care' of people anywhere in the world.

Thus, in response to the second question of this section: The global-as-supraterritorial might not cohere completely with other notions of 'global', which is fine since complete coherence means redundancy. But it may be assembled with other notions [51] or amend apparently conflicting ones [50] to bridge the conceptual gaps and overcome conflicts.

### Reflections on normative objectives, impartiality and objectivity

Some definitions of 'global health' encapsulate the pursuit of normative objectives, claiming validity for those engaged in the field. For example, the goal of 'equitable access to health in all regions of the globe' [13], 'equity in health for all people worldwide' [1] or the reduction of 'global health inequities' [16].

Notably, this is not the case for 'global health' composed of 'global-as-supraterritorial' and 'health' (if the latter is kept undefined). Although there are no normative imperatives attached, the supraterritorial social space is by no means 'neutral', but houses power relations and power struggles, conflict and cooperation, hierarchy and equality [20], equity and inequity.

The normative objectives prevalent in existing definitions [1,13,16] are surely all reasonable and desirable. Their inclusion in 'global health' definitions can be regarded as an applaudable move. It reflects the 'shift from international health' and concerns associated with colonialism toward the 'good intentions' of the actors involved in the field and the solidarity they share with poor, deprived and vulnerable populations worldwide.

But do these normative objectives *de facto *reflect the objectives of actors involved in the 'global health' landscape, i.e. in the supraterritorial health-scape? (The term 'health-scape' is used here following Appadurai's terminology of '-scapes' [51] in order to avoid the conflicting constellation of '*supraterritorial *health *land*scape'.) In other words: does the attachment of normative objectives to definitions of 'global health' constitute an effective 'line of division' between those who share these objectives and those who don't? And if not, which problems arise from ineffective 'lines of division'?

The following section reflects on problems and merits inherent in the different approaches.

#### The entanglement-problems

The attachment of normative objectives to definitions of 'global health' can be seen as 'fact-value entanglement', a shorthand term for the entanglement of facts, conventions and values in the language we use to describe something (see [52] and [53]*(p. 119; 357)*).

These entanglements and 'common goals' in prominent definitions of 'global health' can be seen as important successes in the 'battle of theories' for a just world. But it would be naïve, in our divisive world, to negate the vested and often conflicting interests of different actors, institutions and agencies involved in 'global health'. There is no homogenous 'global health community', but there are many diverse 'global health communities', propelled and formed by their own motivations, values and drivers.

Thus, the author argues that definitions with attached normative objectives do not truly or 'objectively' reflect what the field of 'global health' actually *is*, but rather what it *should *be.

There are two problems with this entanglement:

(i) The first 'entanglement-problem' is that attempts to draw *normative *boundaries around a field often face allegations of 'politicisation' and calls for 'impartiality' and 'objectivity'.

(ii) The second 'entanglement-problem' is that, as soon as the label 'global health' is applied to education, research or practice, these areas not only suggest to be aiming at something 'good', but raise the impression of *per se *doing 'good'. A construct of 'global health', loaded with desirable, normative, but *ineffective *'lines of division' can too easily encapsulate those who *de facto*, viz. in theory do not share, but especially *in practice *do not realise or even actively oppose the normative objectives.

As such, the fate of 'global health' might too easily parallel that of (buzz-) words like 'participation' and 'empowerment', which are meanwhile emptied and partially perverted from their original meaning [54]. Furthermore, worrying developments in the global health-scape can occur under the cover of desirable, *a priori *attached normative 'ideals'. These developments cannot be discussed here in depth for all areas, but shall be outlined in the following.

#### Discrepancies between rhethoric and reality in 'global health'

In 'global health' practice they include, for example, the massive rise of global public-private partnerships [55-57] accompanied by lacking accountability [58,59]; the unequal representation of voices from low- and middle-income countries in decision-making fora [60]; the both democratically and socially unlegitimated dominance of only several players in priority setting [58,61]; or the discrepancy between moral/ethical discourse and real practice in foreign-policy [62].

Examples of worrying arenas erupting in research and education are the 'exploding popularity' of 'global health' in Europe and North America [3,16,63] and the current asymmetrical 'manner of knowledge creation, exploitation, and exchange' [16]. Janes and Corbett thus reminiscently forewarn of 'a new form of colonialism' aimed at satisfying 'the needs of science' [16] rather than the needs of the affected. Massive, unprecedented amounts of funds from private and 'philanthropic' foundations pour into the as yet poorly conceptualised field of 'global health' education [64,65], building on well-attempted but vague and obviously contested [2] definitions [1]. The massive involvement of universities in these areas is pushed not only by motives in line with the (desirable) normative objectives, but apparently also by commercial interests, interests of national security, foreign policy and soft power, blue-washed by human rights rhetoric [3,64]. Too easily may students' highly praised 'interest in global health' [63,64], and with that the potential power of education in this field [8,66-70], be hijacked (via 'new career paths', the creation of 'global health experts' and 'old' re-labelled patterns of sending institutions [64]) and instrumentalized for not totally un-selfish interests (openly [11,64] or less openly [58,59,71-74] communicated).

#### The inherently politicised field of 'global health'

Admittedly, some might regard these developments as not 'worrying'. Taking a view from 'some distance' and attempting not to take side between 'worrying' and 'not worrying' leads to an incontestable, 'objective' (i.e. person-invariant) observation. This observation directly follows from the above-outlined (i.e. from the developments related to the second 'entanglement-problem') and refers to the first 'entanglement-problem':

As for practice, it is obvious and incontestable that policies, programs and actions are negotiated, implemented or opposed in a highly political environment [31,58,75-80], worldwide as well as supraterritorially. 'Impartiality' and 'objectivity' are therefore more often claimed in the areas of research and education, especially in formalised institutes of higher education. As indicated above, however, these institutions *themselves *are nested and operate *within *higher-level social structures *and not *in a political vacuum [81] (*p.376-85*). Setting 'global health' research priorities is as a *political *issue [36,61,82] as determining learning objectives and 'career paths' in 'global health' education [64]. Knowledge, the product of research and substance of education, entails its own political economy in terms of *the way *it is produced and *for what *it is imparted.

At the bottom line: 'global health', research, education and practice are nested in a highly 'politicised' environment, locally as well as supraterritorially. *All *areas accommodate *their own*, but *interdependent *political economy.

So, what follows from this 'objective' observation for the first 'entanglement-problem'? Allegations claiming that a field, which is *inherently *'politicised', *becomes *'politicised' by attaching normative objectives can be regarded as annulled.

The question is not *whether or not *'politicisation' but *which *'politicisation'? Consequently, what remains of the first 'entanglement-problem' as a respectable claim is closely related to the latter question, and is that of 'impartiality' and 'objectivity'. Both of these are not, however, to be blended with '*un- *or *de-*politicisation', as the following illustrates.

#### Impartiality and objectivity in inherently politicised environments

Against the perpetual imminence that universities, education and science become instrumentalized by the politics of reality, Jürgen Habermas, in *Theory and Practice*, pleads for a 'politicisation' of science and education in terms of enhancing mechanisms of *self-reflection *in science [81]. This 'politicisation', he argues, is not only legitimate, but is *the pre-condition of autonomy *in science, which cannot be preserved any more *un*politically today (*ibid., p.385*). The 'politicisation' that he calls for can be regarded as a *self-enlightenment *of sciences as essential prerequisite of 'autonomy' (*ibid.,p.383*) and thus of claims for 'objectivity'.

The ultimate question is thus, how can we 'impartially' and 'objectively' define objectives in a 'politicised' field of 'global health'? Where can this 'self-enlightenment' in the field of 'global health' be expected to come from? These questions overlap with the fields of moral and political philosophy and need thorough consideration of the term 'objectivity'.

In his book *The View from Nowhere*, Thomas Nagel characterises 'objectivity' in the following way: 'A view or form of thought is more objective than another if it relies less on the specifics of the individual's makeup and position in the world, or on the character of the particular type of creature he is' [83].

The merits of seeing objectivity in this way is - as Amartya Sen notes in *The Idea of Justice *[53] - that it focuses on 'position independence' *(ibid., p.157)*, i.e. on the *invariability *of observations in relation to the observers' position. In contrast to Nagel's characterisation, which requires 'a view from nowhere' to achieve 'objectivity', Sen invokes the term 'positional objectivity' (*ibid., p.157*).

This term describes the 'objectivity' of person-invariant but *position-dependent *observations. In an illuminating way (*ibid., p.155-174*), he illustrates (in far more depth and breadth that can be achieved here), how '[..] epistemology, decision theory and ethics all have to take note of the dependence of observations and inferences on the position of the observer' (*ibid. p.157*). The quality of observations that are made is thus dependent on the observer's positional characteristics (e.g. knowledge, education, physical abilities). That is, any person occupying the same position or facing the same conditions would come to the same quality of observations (therefore person-invariant and not equal to 'subjective').

This kind of 'objectivity', however, is prone to 'objective illusions'. These can be understood as 'a positionally objective belief that is, in fact, mistaken in terms of transpositional scrutiny' (*ibid., p163*). That means that parochial (position-dependent) 'objective' observations, beliefs or decisions need transpositional correspondents, to weigh whether the observation is indeed 'objective' or an 'objective illusion' (*see for example 'Health, Morbidity and Positional Variations' in *[53], *p.164*). To enable transpositional scrutiny, Sen (*by following Adam Smith's notion of the 'impartial spectator', see *[84]*cited in *[53], *p.124*), calls for processes of 'open impartiality' (*ibid., p.123*). In these processes, impartial assessments not only *can *but often *must *invoke judgments and reasoning from outside a particular group (*ibid., p.123*), in order to overcome the confounder of 'objective illusions' and parochial bias.

In a nutshell, Sen regards public reasoning and 'democratisation', in terms of political participation, dialogue and public interaction, as means to reach this end (*ibid., 326*), providing grounded reasoning for his views [53].

Thus, we may regard 'democratisation' in this sense as enabler of 'open impartiality' which, in the special case of science and education, would fulfil Habermas's prerequisite 'politicisation' to *progress *toward a state of 'self-enlightenment' *(though Habermas's view of 'democratisation' in *[81]*was of more institutionalised nature*).

#### Impartiality and objectivity in 'global health'

Back to the remnant claims of the first 'entanglement-problem': what is the consequence of 'positional objectivity', 'objective illusions' and 'parochial bias' for the claim that goals in the highly 'politicised' field of 'global health' should be set 'impartially' and 'objectively'?

With respect to the 'politics of the possible' (i.e. the plurality of possible choices and plurality of reasons for the choices) in today's global health-scape [85], the elements of congruence which survive open, impartial reasoning (e.g. via public debate, interaction and participation by people, especially from beyond 'epistemic communities' [16]) can thus be regarded as legitimated (i.e. accepted, ideally but not necessarily, by all) to form the basis of a partial ordering [53] (*pp.394-415*). The concept of universal human rights is such an example of 'partial orderings', obtained and accepted via long processes and struggles of 'open impartiality' (*ibid; pp.355-388*).

Claims of 'partial orderings' to enhance objectives, such as health equity and solidarity, dominate in contemporary definitions of 'global health' [1,13,16]. These claims can also be regarded as the congruent, surviving elements of continued - 'historical' [86,87] and contemporary [88,89] - public reasoning worldwide as well as supraterritorially. These objectives, although of normative and 'partial' nature, can be regarded as produced by processes of 'open impartiality' with minimised possibility of 'parochial bias'.

These claims are, in terms of Nagel's 'objectivity', more objective (i.e. less position-dependent) than decisions made in and priorities set by closed 'epistemic communities' or decision-making fora, in which 'transpositional' views are either neglected or underrepresented.

As a consequence from the above, for the first 'entanglement-problem': to 'objectively' determine in which direction 'global health' research, practice and education *should *go, there might be no other way than 'politicisation' of the field through 'open impartiality', i.e. via public reasoning, political participation, dialogue and public interaction [53] including, if necessary, social mobilisation or resistance [58].

As for the second 'entanglement-problem': in order to avoid that definitions of 'global health' blur the discrepancy between 'reality' and 'ideals', these definitions should abstain from attaching normative objectives *a priori *and factually describe what the field *is*, not what it ideally *should *be.

Normative objectives are highly important to frame the debate and to hold all actors accountable to work toward the achievement of obtained 'partial orderings'. However, as long as normatives neither constitute effective 'lines of division' nor effective enforcement mechanisms [62], framing should not be done via 'global health' definitions. Framing the debates via 'health' (e.g. health as defined above vs. as absence of disease or vs. a commodity) is strong enough, since health *is *(not *should be*) a universal human right.

#### Resolving the entanglement-problems

The following shows how this solves our entanglement-problems: health as a human right (HHR) as imperative for partial orderings is factually binding and enforceable (though yet with limited effectivity) via international law [90] but not yet fulfilled and violated globally (*read: worldwide and supraterritorially*).

Whether the prevailing culture of 'global health' (however defined) in research, education or practice fulfils the then resulting necessary steps and entailed normative objectives to fulfil HHR cannot be defined *a priori*. The question remains subject to empirical and critical, self-reflective scrutiny of the 'global health community'.

In this context, the major force of the definition of 'global health', consisting of global-as-supraterritorial and 'health' (as defined above) would lie in drawing our view:

(i) on the actual architecture of the 'global' social space, defined as links between the social determinants of people's health anywhere in the world and

(ii) on health as a social, economic, political issue and as a fundamental (but non-fulfilled) human right.

Education and research in 'global health' thus implies an engagement with the question of how processes of acquiring, distributing and exercising social power in creating these links reduce or maintain violations of HHR. Also, practitioners (organisations, interest groups, politicians etc.) who constitute, produce or influence these links find themselves in a social space, where their actions mean progress or regress toward fulfilling HHR worldwide.

By being disengaged from normative objectives, but not disengaged from the above imperative for partial orderings (HHR), this definition would draw the attention on the bare political economy in and of 'global' (read: supraterritorial and worldwide) health actions and actors.

With global-as-supraterritorial, the 'global' in 'global health' is both neutral (i.e. not loaded with normative/ethical claims) and un-neutral enough (i.e. not de-politicised) to serve as a self-reflective, un-euphemistical approach to practising, understanding and teaching the inherently 'politicised' nature of 'global health'. The field is about building and re-building, researching and analysing, teaching and learning the links between social determinants of people's health anywhere in the world.

### Notes on definitional paradoxes and the omnipresence of ambiguity

Before closing, it is necessary to reflect about three paradoxes involved in defining 'global health'. Definitions draw boundaries around institutional fields and are always simultaneously inclusive and exclusive; while some organisations, practitioners and practices will be inside, others will be outside those boundaries.

Thus, the first paradox in attempts to define 'global health' is reflected in the effort to draw a boundary around a field, which has emerged due to its 'boundary-transcending' character.

The second paradox is that, by *confining *the focus of 'global health' on global-as-supraterritorial, the field is *widened *towards an object, which enables health related disciplines to engage therein while *avoiding redundancy *and conflicts. The field as such does not become a 'discipline', but rather a field highly inclusive of all health researchers, educators and practitioners from different backgrounds who focus on the supraterritorial links between the social determinants of health anywhere in the world.

Thirdly, despite the dialectics of the presented concepts of the 'global', these are not exclusive of, but rather complementary to each other. Global-as-supraterritorial always needs two other 'global' concepts, namely the *worldwide *as well as the *holistic*. We need more *worldwide *impacts to be considered, for example in decision-makings on supraterritorial 'levels', and at the same time need more global (*read: supraterritiorial*) perspectives in health research, education and practice worldwide to meet global (*read: universal and/or supraterritorial*) challenges. Similarly, global-as-supraterritorial is useless when regarded in isolation and in a reductionist way: this concept needs to consider the influences on health on international, national, regional, local, community, individual and the biomedical level to assemble the 'whole picture' in a global (*read: holistic*) concept to improve health worldwide in the final step.

Due to these three paradoxes, only positive definitions of what 'global health' includes seem to be possible, while negative definitions of what it does not include are highly questionable and depend on the concept of 'global' and frames of 'health'. That means that all of the above definitions are applicable in *their particular context*. Also, we have to accept that 'global health' not *'is'*, but *can *be 'public health' [2] depending on the chosen concept of 'global' - but it can (and should) be more depending on the same.

#### The omnipresence of ambiguity

By this stage, some might be disappointed by the degree of ambiguity they faced throughout the manuscript: the 'local' as both territorial *and *supraterritorial; the 'global' as supra-territorial but not *un-*territorial; pursuit of 'objectivity' through 'politicisation' and 'democratisation'; and finally 'definition paradoxes'.

As one of the reviewers (JÖ) of this manuscript noted, this is inevitable when attempting to describe a vaguely defined field ('global health') with an ambiguously connotated phenomenon ('globalization'). In this context (i.e. in the 'globalization' discourse), Van Der Bly bemoans the triumph of ambiguity in social science [91] and lauds the (apparently) clear definition of 'globalization' by economists. Referring to the World Bank, she states that '[e]conomists seem to have succeeded in reaching more or less a commonly accepted definition of globalization, namely as international economic integration that can be pursued through policies of 'openness', the liberalization of trade, investment and finance, leading to an 'open economy'' [91].

Most of her substantive critique on definitions of 'globalization' can be allayed by reading Scholte [20]. But the above quote reveals an interesting phenomenon, overseen by Van Der Bly and maybe by readers of this manuscript, who feel uncomfortable with ambiguity and the entailed 'uncertainty'. This phenomenon could be called the *omnipresence *of ambiguity.

Take the above statement as example: what is called 'policies of openness [..] leading to an 'open economy'' is not achieved by nature. The economic 'openness' is achieved by hard law and binding *regulations*, which *restrict *regulatory space (see Table III in [25] for examples of WTO *regulations *and loss of domestic *regulatory *space). So the 'globalization' of 'policies of openness' are, at the same time, the 'globalization' of 'policies of restrictions'. Without global restrictions or regulations, there is no global 'openness'. The apparently clear definition is revealed to be an ambiguous one. So, are ambiguous statements (or definitions, theories, frameworks, etc.) generally imprecise?

In this context, Amartya Sen defends the methodological point that '[..] if an idea has an essential ambiguity, a *precise *formulation of that idea must try to *capture *that ambiguity rather than lose it.' [92]*(p.48-9; emphasis in original)*.

As such, ambiguity is not at all a *carte blanche *for unreflexive pluralism. Beyond doubt, the plausibility of different concepts and meanings of the 'global' in different contexts is itself an argument against insisting unconditionally on *one *concept. But the inherent ambiguities, precisely captured and uncovered in this manuscript, call especially on researchers and educators to be clear about which 'global' they mean when researching or teaching 'global health'.

Being aware of the ambiguities and controversies presented in this manuscript is necessary for not getting trapped in discussions on 'ins' and 'outs', but to research, teach and work together towards better health for all globally (*read: as you want, but be specific about it*).

#### Implications from the proposed concept

The implications of global-as-supraterritorial are not only of theoretical interest, they also have some practical importance for the areas of research, education and practice. One of the anonymous reviewers of this manuscript has argued that 'the definitions of global would be different for each of these three areas'. Acknowledging this fact, the author believes that the debate addresses questions with relevance at the interface of all these three areas. Whether or not the proposed concept of 'global-as-supraterritorial' can be a *common denominator *for all three areas (or for everyone involved in 'global health') is certainly a subject of debate (and as stated above, not necessarily given). The concept, however, can be a *starting point *for discussing these differences in all three areas.

**Figure 3:**
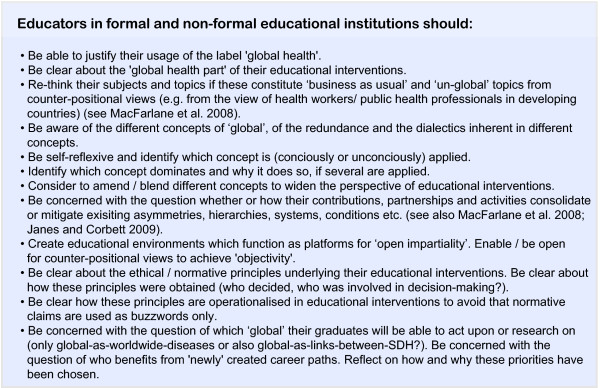
**Suggestions for educators involved in global health education**.

Some concrete suggestions are made in Figure 3 for educators involved in 'global health education' on how to move forward self-critically. Implications and challenges for 'global health' research can be found in the literature at the interface of social determinants and globalization [25,37,38,40,93-96]. These are not 'new' in light of the proposed concept, but gain momentum greatly from it in terms of rational validity when we speak about 'global health' priorities [36,82,97].

## Summary

This manuscript has argued that current definitions of 'global health' lack specificity about the term 'global'. It has shown that common understandings of the element as *worldwide *or as *transcending national boundaries *are either misleading or produce redundancy with other health related fields and disciplines. 'Holistic' notions are highly comprehensive and inclusive, but entail evaluation problems without being defied from producing redundancy (see Additional file 1).

With the concept of global as *supraterritorial *(Figure 1), the field of 'global health' can be practiced, taught and analysed by representatives of the 'public health' as well as the 'international health' community. Globality in this context refers to supraterritorial links between the social determinants of health *anywhere *on earth, while avoiding redundancy with disciplines that focus on health as a social, economic and political issue or as a human right (Figure 2). This has been shown by means of the examples of malnutrition; HIV, tuberculosis & malaria; and maternal mortality (see Additional file 1). As such, globality adds to the complexity of social space. It links the social determinants of health and thus people horizontally *anywhere *in the world and impacts on them (people and their SDH) through complex pathways.

The author has put the proposed concept in context with other views on 'global' and 'local', concluding that the 'global-local-relationship' inherent in the proposed concept coheres (not with all but) with influential anthropological and sociological views despite the use of different terminology.

Further attention has been paid to problems that follow from normative objectives *a priori *attached to definitions of 'global health'. The manuscript argues that definitions should abstain from attaching normative objectives *a priori *and factually describe what the field *is*, not what it ideally *should *be. The author argues for 'democratisation' and 'politicisation' of the field, to obtain and maintain desirable underlying normative objectives of the field. The responsibility of the 'global health community' is then to assess via empirical and critical, self-reflective scrutiny whether the *prevailing *culture of 'global health' research, education or practice meets these objectives. The proposed concept, linked with health as a human right (HHR), has been argued to be suitable also for this purpose.

Social innovations are unlikely to evolve if 'global health' becomes or remains a cosmetic re-labelling of old patterns, objects and interests. Rather, by focussing on the globality of the social determinants of health and the power relations in global (*read: supraterritiorial*) social space, professionals involved in health research, education or practice can contribute to analysing, developing and teaching more innovative strategies worldwide toward fulfillment of HHR.

The paradoxes involved in attempts to define 'global health' finally demonstrate that the dialectics inherent in the different concepts of the term 'global' are not exclusive, but rather complementary to each other. The author has captured other ambiguities several times throughout the manuscript and argues against insisting unconditionally on one concept of 'global'. Ambiguity, however, is not at all regarded as a *carte blanche *for unreflexive pluralism, but calls especially on researchers and educators (Figure 3) to be precise in the usage of their terms. It is unavoidable that terms have different meanings to different societal actors. The concept presented in this debate, however, has provided a rational validation for arguments to preserve the rhetorical power of the descriptor 'global health' for more 'innovative' forms of research, education and practice compared to common 'global health' discourses.

With this manuscript the author hopes to provoke further debates on the crucial issue of conceptualising the field of 'global health'.

## Competing interests

### Financial competing interests

The author declares that he has no conflict of interest. The article processing charge was covered by the Institute for Social Medicine, Epidemiology and Health Economics, Charité - University Medical Center Berlin, Germany.

### Non-financial competing interests

This manuscript has been produced as part of the research thesis of KB to gain a scientific degree *(Dr.med) *at the Institute for Social Medicine, Epidemiology and Health Economics at the Charité - University Medical Center in Berlin, Germany.

The concept of global health described in Figure 1 is adapted from the social determinants of health model of Dahlgren and Whitehead and the globalisation and health model of Huynen and colleagues. It has originally been developed and used by KB and Dr. Peter Tinnemann (Dept. for International Health, Institute for Social Medicine, Epidemiology and Health Economics, Charité - University Medical Center Berlin, Germany) as part of a framework to assess global health in medical education in Germany.

## Authors' contributions

KB was responsible for content, conception and design of the manuscript. He drafted and edited the manuscript and produced the figures.

## Authors' information

KB is a 6^th ^year medical student (University of Frankfurt/Germany), undergoing a research fellowship at the Dept. for International Health at the Charité - University Medical Center in Berlin, Germany. He has been involved in student organisations on national (Globalisation and Health Initiative, bvmd e.V.) and international (Think Global Initiative, IFMSA) levels. Currently, KB is a member of the 'Young Professionals Commission', an informal advisory group to the 'Global Commission on Education of Health Professionals for the 21^st ^century' http://www.globalcommehp.com; and an independent advisor to a commission (*'Ausschuss Nachwuchsförderung') *of the German Society for Tropical Medicine and International Health (*Gesellschaft für Tropenmedizin und Internationale Gesundheit*) aimed at capacity building.

## Supplementary Material

Additional file 1 **Table 1-The dialectics of global**. Click here for file
