# Supplementary material for: Rethinking the 'global' in global health: a dialectic approach
Source: Global Health. 2010 Oct 28;6:19. doi: 10.1186/1744-8603-6-19 (PMC2987787; doi:10.1186/1744-8603-6-19)
Supplement: Additional file 1 — Table 1-The dialectics of global. [file 1744-8603-6-19-S1.PDF]

Concepts of 'global'	Malnutrition	HIV, Malaria & Tuberculosis	Maternal mortality	Implications
**Global-as-worldwide / universal**	Overnutrition and obesity become a global health problem. The field focuses on its determinants worldwide, while undernutrition remains a medical and public health problem of low-and-middle-income countries.	HIV and tuberculosis become global health problems and the field focuses on its worldwide prevalent determinants, while Malaria remains a medical and public health problem of the 'tropics'.	Maternal mortality remains a medical and public health problem of low-and-middle income countries.	This concept allows some issues to become objects of global health, because they constitute problems worldwide or are 'universal' health problems. The concept does not legitimate that problems with endemic character or highly unequal burdens across the world are declared as global health problems. **In cases, in which the concept of 'universality' applies, it produces high redundance with the object of public health.**
**Global-as-transcending-national-boundaries**	Malnutrition remains a medical and public health problem. The carriers which transport risk factors and lifestyles across more than one country and lead to malnutrition, e.g. international trade, become the object of global health.	HIV, tuberculosis and malaria become global health problems (because they can be transmitted and transcend national boundaries) as well as any related determinants which influence more than one country.	Maternal mortality remains a medical and public health problem. Transnational (health) policies affecting maternal mortality in more than one country become the object of global health.	All communicable diseases become global health issues *per se*, while all non-communicable diseases do not. All determinants affecting *more than one country* (i.e. transcend at least one national border) become the object of global health. **This concept produces high redundance with the object of international health.**
**Global-as-holistic**	Genetic constituencies, physical activity, lifestyles, food production, food security and availability, national -, international and worldwide trade, poverty, disasters etc. become the object of global health in this context.	Individual behaviour, biomolecular aspects of transmission and infection, access to medicines, prevention, health promotion, research and development, poverty, etc. become the object of global health in this context.	Biomedical causes of maternal mortality (infections, heamorraghe, eclampsia), physical and social access to health care, gender issues, socio-economic inequalities and inequities with and between countries, the MDGs, global health initiatives etc. become the object of global health in this context.	With this concept every health issue has a global health aspect, since it is concerned with *all* influences.<br>**While this concept might be the most comprehensive at the first glance, it produces high redundance with the field of biomedicine, public health and international health, while 'global' influences are still conceptualised as 'universal'.**

**Global-as-supraterritorial**	Malnutrition remains a medical and public health problem. The social links between people anywhere in the world in the context of malnutrition, i.e. supraterritorrial influences on the (territorial) social determinants of malnutrition become the object of global health; such as International Agreements (e.g. Agreement on Agriculture of the World Trade Organization), the rise and role of transnational food corporations, subsidies for agrofuel and energy consumption, stock markets and food speculations, etc.	HIV, tuberculosis and malaria remain a medical and public- or international health problem. Supraterritorrial influences on the (territorial) social determinants of HIV, malaria or tuberculosis become the object of global health; e.g. Trade Related Aspects of Intellectual Property Rights (TRIPS), the 10/90 gap, policies of transnational drug companies, global health initiatives and intended / unintended impacts on downstream determinants etc.	Maternal mortality remains a medical and public – or international health problem. Supraterritorrial influences on the (territorial) social determinants of high maternal mortality become the object of global health; e.g. the role and interplay of global health initiatives in reducing / increasing safe deliveries and social-, physical or financial access to health care or gender inequities; the impacts of the world food and economic crisis on the determinants of maternal mortality; policies affecting emigration of health care professionals etc.	With this concept, diseases and illnesses remain medical and public health or international health problems.<br><br>The disease specific aspects rather become symptoms of underlying structural determinants *AND* their supraterritorial links.<br>**This concept applied in global health studies, research and practice produces new knowledge, insights and solutions.**
